# The prognostic significance of DAPK1 in bladder cancer

**DOI:** 10.1371/journal.pone.0175290

**Published:** 2017-04-07

**Authors:** Jian-Yun Xie, Peng-Chen Chen, Jia-Li Zhang, Ze-Shou Gao, Henrique Neves, Shu-Dong Zhang, Qing Wen, Wei-Dong Chen, Hang Fai Kwok, Yao Lin

**Affiliations:** 1 Department of Urology, The Affiliated People’s Hospital of Fujian University of Traditional Chinese Medicine, Fuzhou, Fujian Province, People’s Republic of China; 2 College of Life Sciences, Fujian Normal University, Fuzhou, Fujian Province, People’s Republic of China; 3 Faculty of Health Sciences, University of Macau, Avenida da Universidade, Taipa, Macau SAR; 4 Northern Ireland Centre for Stratified Medicine, University of Ulster, C-TRIC Building, Altnagelvin Hospital Campus, Glenshane Road, Londonderry, United Kingdom; 5 Centre for Cancer Research & Cell Biology, School of Medicine, Dentistry and Biomedical Sciences, Queen’s University Belfast, Belfast, United Kingdom; University of Michigan, UNITED STATES

## Abstract

Bladder cancer is one of the leading causes of cancer-related death in men, however, there was only limited effective treatment for invasive bladder cancer. DAPK1 has been shown to play important role in apoptosis and autophagy to suppress cancer progression. Previous results have shown that DAPK1 promoter was hypermethylated in the majority of bladder cancer specimens, however, the prognostic significance of DAPK1 in bladder cancer has yet to be demonstrated. In the present study, we found that DAPK1 expression was negatively associated with tumor stage and a low level expression of DAPK1 in bladder cancer specimens were associated with shorter survival in bladder cancer patients in 3 independent bladder cancer datasets (n = 462). Further investigation showed that FGFR3 knockdown resulted in downregulation of DAPK1 in bladder cancer cell line, suggesting that FGFR3 may be an upstream factor of DAPK1. Further analysis of the 3 independent bladder cancer datasets have identified ACOX1, UPK2, TRAK1, PLEKHG6 and MT1X genes had their expression significantly correlated with that of DAPK1. Knockdown of DAPK1 in bladder cancer T24 cells resulted in downregulation of ACOX1, UPK2 and TRAK1. Interestingly, TRAK1, by itself, was a favorable prognostic marker in the 3 independent bladder cancer datasets. Importantly, by using connectivity mapping with DAPK1-associated gene signature, we found that vemurafenib and trametinib could possibly reverse DAPK1-associated gene signature, suggesting that inhibition of Raf/MEK pathway may be a potential therapeutic approach for bladder cancer. Indeed, treatment of vemurafenib in T24 bladder cancer cells resulted in upregulation of DAPK1 confirming our connectivity mapping, while knockdown of DAPK1 resulted in reduced sensitivity towards inhibition of Braf signaling by vemurafenib. Together, our results suggest that DAPK1 is an important prognostic marker and therapeutic target for bladder cancer and have identified possible therapeutic agents for future testing in bladder cancer models with low DAPK1 expression.

## Introduction

Bladder cancer is the 6^th^ most common cancer and the 9^th^ leading cause of cancer-related death worldwide in men [1]. Although the understanding of bladder cancer has improved dramatically in the last decade, novel treatment that could potentially improve patient outcome has just recently been discovered [2–4]. Bladder cancer can be divided into muscle invasive and non-muscle invasive, in which muscle invasive bladder cancer has poor prognosis compared to non-muscle invasive bladder cancer [5]. Recent advances in expression profiling suggest that bladder cancer can be further divided into various subtypes, which may require different disease management and treatment in the future, depending on its molecular characteristics [6].

Death-Associated Protein Kinase 1 (DAPK1) is a member of the serine/threonine kinases that could act as a tumor suppressor in relation to its function to regulate apoptosis and autophagy [7]. We have previously shown that a germ line mutation in death domain of DAPK1 could diminish its function in promoting apoptosis [8] and the protein stability of DAPK1 is regulated by both the TSC1 [9] and its own splice variant [10], suggesting that DAPK1 expression was post-translationally regulated. On the other hand, data were also available showing that DAPK1 is regulated at the transcriptional level via its promoter methylation. DAPK1 promoter has been shown to be hypermethylated in bladder cancer [11, 12]. Demethylating agents have been shown to reverse the hypermethylation status of DAPK1 promoter leading to re-expression of DAPK1 mRNA resulting in retarded growth of bladder cancer cell lines [13]. In a study with 34 bladder cancer samples, the correlation between DAPK1 mRNA expression and tumor staging and grading could not be established [14].

In this study, we aimed to investigate the prognostic significance of DAPK1 expression in bladder cancer, identify genes that are co-regulated with DAPK1 in bladder cancer and identify small molecules that could reverse the DAPK1-associated gene signature.

## Materials and methods

### Extraction of clinical and microarray gene expression data from bladder cancer patient datasets

A search was performed using “bladder cancer or Urothelial cancer in Gene Expression Omnibus database. Four datasets with more than 130 cancer patients in a single microarray platform, GSE13507 (n = 165) [15, 16], GSE32548 (n = 131; Survival data was not available in this dataset) [17], GSE32894 (n = 308) [18] and GSE48277 (n = 142) [19], were identified (S1 Fig). In addition, another dataset GSE41035 with modification of FGFR3 gene in bladder cancer dataset was also used in the current study to test the association between FGFR3 and DAPK1 [20]. The GEO website has standardized URLs for its individual dataset, the files in gzip format were unzipped to the tab-delimited text format, which contain detailed clinical information for statistical analysis. R scripting was used to extract the expression values of probesets from genes of interest and the clinical data from the data matrixes downloaded from the GEO database was then combined for analysis.

### Statistical analysis

All statistical analyses were performed using SPSS19.0. The associations between gene expression and clinicopathological parameters were tested by ANOVA with post-hoc Bonferroni test for multiple comparisons. Tumors with T-stage of T2 or above were classified as having a high T-stage. The associations between gene expression and survival were estimated by Kaplan-Meier survival analysis and statistically tested by log-rank test. The associations between expression levels of genes were analyzed by Spearman’s rank test. Univariate Cox-regression was used to test the correlations between gene expression levels and survival.

### Identification of DAPK1 co-expressing genes

Patients were stratified into four groups based on the expression levels of DAPK1. The gene expression patterns of patients in DAPK1-low subgroup and those in the DAPK1-high subgroup were compared. Probesets that were differentially expressed between these two subgroups were identified by 2-sample Welch’s T-test. This test was used to avoid the type I error due to unequal variances of the values of probesets between subgroups. Briefly, a Welch’s t test was applied to each probeset corresponding to a certain gene in the data matrix using our own Java application MyStats. *P* values and the differential expression in fold changes for all the probesets were generated as tab-delimited worksheets of Excel for further analysis. The genes were ranked based on the p value for all the probesets available for a particular gene and the top 5 genes were extracted from the GEO database for further analysis.

### Identification of potential therapeutic small molecule for bladder cancer patients overexpressing DAPK1 through connectivity mapping

Gene expression connectivity mapping was performed using Statistically Significant Connection’s Map (sscMap) to identify candidate small molecule compounds that may reverse the reduced expression of DAPK1 and its associated gene expression signature [21–23]. The relevant probes were input to the Java application sscMap [23] as a query signature, and its association with the 6000 gene expression profiles generated by treating cancer cells with over 1000 small molecules were compared. The gene signature perturbation procedure, which increases the specificity of the output results, was applied as previously described [24]. All the small molecular compounds that were negatively associated with the input were sorted and ranked by their *p*-value, perturbation stability and standardized connection score. The *p*-value that was considered significant was set at a stringent threshold (p = 1/1309), ensuring that the results generated by sccMap would yield a maximum of one false positive small molecule over the 1309 small molecules tested in the sccMap [24].

### Construction of stable cell lines

Human transitional carcinoma cell lines T24 derived from bladder epithelial tissue was purchased from the Institute of Biochemistry and Cell Biology at the Chinese Academy of Sciences (Shanghai, China). T24 cell lines was maintained in RPMI 1640 (Invitrogen Corp., Carlsbad, California) containing 10% fetal bovine serum (FBS) (Equitech-Bio, Ingram, Texas), 100 units/ml penicillin G and 100 μg/ml streptomycin (Invitrogen Corp.). Cells were incubated at 37°C in a humidified incubator with 5% CO2. To create DAPK-knockdown bladder cancer cells, lentiviral infection was performed. Briefly, control and DAPK-knockdown cell lines were established utilizing lentivirus particles containing scramble shRNA (SHC016V) or DAPK-target shRNA (SHCLNV-NM_004938) from Sigma (USA); Sequences of the shRNA are listed in S1 Table

Bladder cancer cells were seeded onto plates and infected with lentiviral particles in 1640 containing 8 μg/ml polybrene (Sigma). One day after the incubation, the media containing lentivirus was changed with the growth media, and the cells were incubated for another day. Puromycin (Invitrogen) at 0.4 μg/ml was added to each well to select for cells efficient for DAPK depletion. The shRNA sequences are shown in S1 Table.

### Western blot

Cells were homogenized in 100~200μl of radioimmunoprecipitation assay (RIPA) lysis buffer (Beyotime, Shanghai, China) containing protease inhibitors (Roche, Basel, Switzerland) and placed at 4°C for 30 mins. Protein concentrations were measured by Micro BCA protein assay kit (Pierce Biotechnology). Protein was resolved on sodium dodecyl sulfate-polyacrylamide (SDS) gel and subsequently transferred to a nitrocellulose membrane (Life Technologies, Carlsbad,CA, USA). The nitrocellulose membrane was then incubated with the primary antibodies and with the horseradish peroxidase (HRP)-conjugated goat anti-rabbit or anti-mouse secondary antibodies, with phosphate buffer saline (PBS) buffer washes in between. The anti-DAPK and anti-GAPDH antibodies were from Cell Signaling Technology (Beyotime, Shanghai, China). The secondary antibodies were from Sigma-Aldrich (St. Louis, MO).

### SRB assay

Cells were first seeded in 6cm dishes and allowed to grow for 3 days. Then for SRB assay, stable cell lines were seeded at a density of 2500 cells/well into 96-well plates respectively. Next cells continued to grow with vemurafenib for another 72h. After culture, total cell amount were detected using SRB assay. At the end of the respective incubation time, cells were fixed by the addition of 10% trichloroacetic acid and incubated for 1 hour at 4°C. Plates were washed in tap water five times and allowed to dry. Cellular protein was stained by adding 4% SRB in 1% acetic acid and incubated at room temperature for 30 minutes. Excess SRB was removed by washing the wells with 1% acetic acid and remaining SRB was solubilized in 10 mM Tris-base unbuffered. Absorbance was determined on a microplate reader(BiOTeK, USA) at a wavelength of 510 nm. Data were obtained from three wells per condition.

### RNA extraction, transcription and real-time PCR

The mRNA from cell lines was extracted using the EastepTM Total RNA Extraction Kit (Promega, Beijing, China) and reverse transcribed using the GoScript™ Reverse transcription system (Promega, Madison, USA) according to the manufacturers’ instruction. Amplification reactions were performed in ABI 7500 real-time PCR system (ABI, America), and PCR was performed using GoTaqTM qPCR Master Mix according to the manufacturers’ instruction. The Real-Time PCR primers for DAPK1, UPK2, TRAK1, ACOX1 and Glyceraldehyde-3-phosphate dehydrogenase are listed on S2 Table. PCR reaction conditions were as follows: 95°C for 2 min, 40 cycles at 95°C for 15 s and 60°C for 1 min. The cDNA from cells were used as positive control and standard curve.

## Results

### The association between DAPK1 mRNA expression and clinicopathological parameters

**Fig 1:**
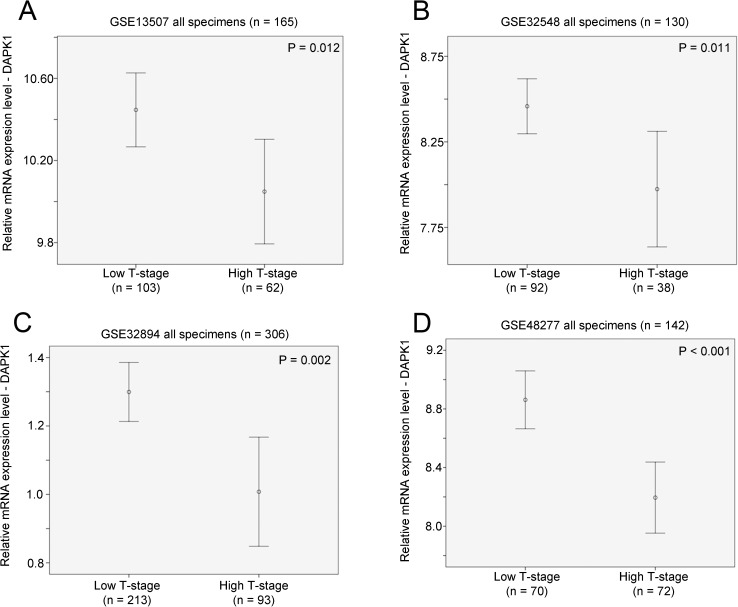
The association between mRNA expression of DAPK1 and T-stage. Error bar plots for DAPK1 expression against high (T-stage ≥ 2) or low (Ta or T1-stage) in bladder cancer cohort (A) GSE13507 (n = 165), (B) GSE32548 (n = 130), (C) GSE32894 (n = 306) and (D) GSE48277 (n = 142). We also tested whether DAPK1 expression was associated with other clincopathological parameters, and we found that DAPK1 expression was not associated with tumor grading, N-stage and M-stage in all the four datasets analyzed in the current study.

There were 165 bladder cancer specimens with available clinicopathological and survival information in the GSE13507 bladder cancer patient cohort. DAPK1 expression was significantly higher in bladder cancer with a Ta or T1 stage compared to those with T2 stage or above (p = 0.012; Fig 1A). Similarly, in GSE32548 dataset, there were 131 bladder cancer patients with available clinicopathological information with one patients had missing T-stage information. In this patient cohort, DAPK1 expression was significantly lower in bladder cancer specimens with a T2 stage or above compared to those with Ta or T0 stage (p = 0.011; Fig 1B). Similar results were also observed for GSE32894 bladder cancer cohort, in which, there were 308 bladder cancer patients with available clinicopathological and 306 of these patients had available information on their T-stage. In this patient cohort, DAPK1 expression was significantly lower in bladder cancer specimens with a T2 stage or above compared to those with Ta or T1 stage (p = 0.002; Fig 1C). Lastly, for the 142 patients with available information on staging in GSE48277, a significant association between high DAPK1 mRNA expression and Ta/T1 stage was also observed (p < 0.001; Fig 1D).

**Fig 2:**
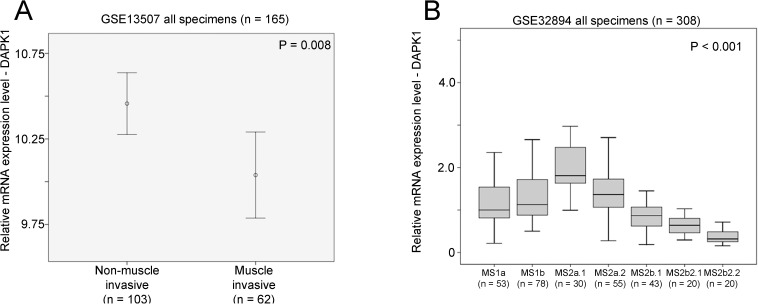
The association between mRNA expression of DAPK1 with muscle invasiveness and gene signature subtype. (A) An error bar plot for DAPK1 expression in non-muscle invasive and muscle invasive types of bladder cancer cohort GSE13507. (B) A box plot for DAPK1 expression in bladder cancer with different molecular subtypes

However, although only available in GSE13507 patient cohort, low level expression of DAPK1 was significantly associated with muscle invasive bladder cancer specimens compared to non-muscle invasive bladder cancer specimens (p = 0.008; Fig 2A). Molecular signature was available in GSE32894 dataset, and we also found that DAPK1 expression was significantly lower in the more aggressive subtypes of bladder cancer, Urobasal B (MS2b2.1) and SCC-like (MS2b2.2) subtypes (p < 0.001; Fig 2B).

### The association between DAPK1 mRNA expression and patient survival

**Fig 3:**
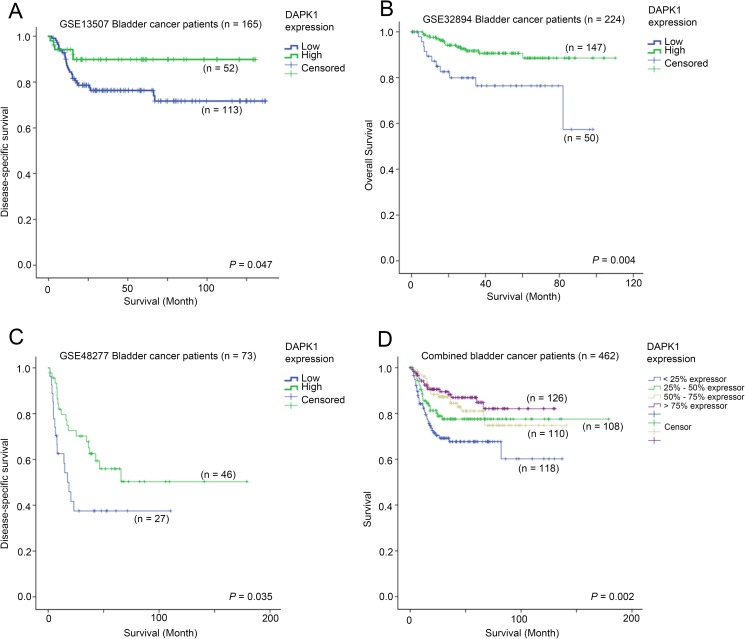
The association between mRNA expression of DAPK1 and bladder cancer patient survival. Kaplan-Meier plots for survival with different level of DAPK1 expression in (A) GSE13507 (n = 165; all patients with survival data available), (B) GSE32894 (n = 224; patients with available survival data), (C) GSE48277 (n = 73; patients with available survival data) and (D) combined cohort.

Three out of the four datasets analyzed in the current study had available information on patient survival data. The association between DAPK1 expression and patient survival was tested by Kaplan-Meier analysis compared by log-rank test. In GSE13507, we found that patients whose tumors had the top 25% expression of DAPK1 had a significantly longer disease-specific survival time (mean = 118 months, 95% CI = 109–128 months) compared to other patients whose tumors expressed a lower level of DAPK1 (mean = 104, 95% CI = 94–115 months, p = 0.047; Fig 3A). In GSE32894, patients whose tumors expressed DAPK1 at bottom 25% level had a shorter survival time (mean = 75 months, 95% CI = 64–75 months) compared to those patients whose tumors expressed DAPK1 at a higher level (mean = 101 months, 95% CI = 96–106 months, p = 0.004; Fig 3B). In the 73 patients whose survival data were available in GSE48277 patient cohort, patients whose tumors had DAPK1 expression at the bottom 25% percentile had a significantly shorter disease-specific survival time (mean = 48 months, 95% CI = 29–67 months) compared to those patients whose tumors had a higher expression of DAPK1 (mean = 103 months, 95% CI = 77–129 months, p = 0.035; Fig 3C). When combined the three datasets together, we found that patients whose tumors expressed DAPK1 at bottom 25% percentile had a significant shorter survival time (mean = 92.544; 95% CI = 79–106) compared to those patients whose tumors expressed DAPK1 at a higher level (mean = 146; 95% CI = 137–154; p = 0.002; Fig 3D). By Cox regression analysis, we found that patients whose tumors expressed DAPK1 at a higher level had a lower risk of death or progression. Comparing to < 25% DAPK1 expressors, 25% - 50% DAPK1 expressors had a 38% lower risk (HR = 0.62, 95% CI = 0.36–1.05, p = 0.075), 50% - 75% DAPK1 expressors had a 53% lower risk (HR = 0.47, 95% CI = 0.27–0.83, p = 0.009), while > 75% DAPK1 expressors had a 63% lower risk (HR = 0.37, 95% CI = 0.20–0.67, p = 0.001).

### The association between FGFR3 and DAPK1 expression

**Fig 4:**
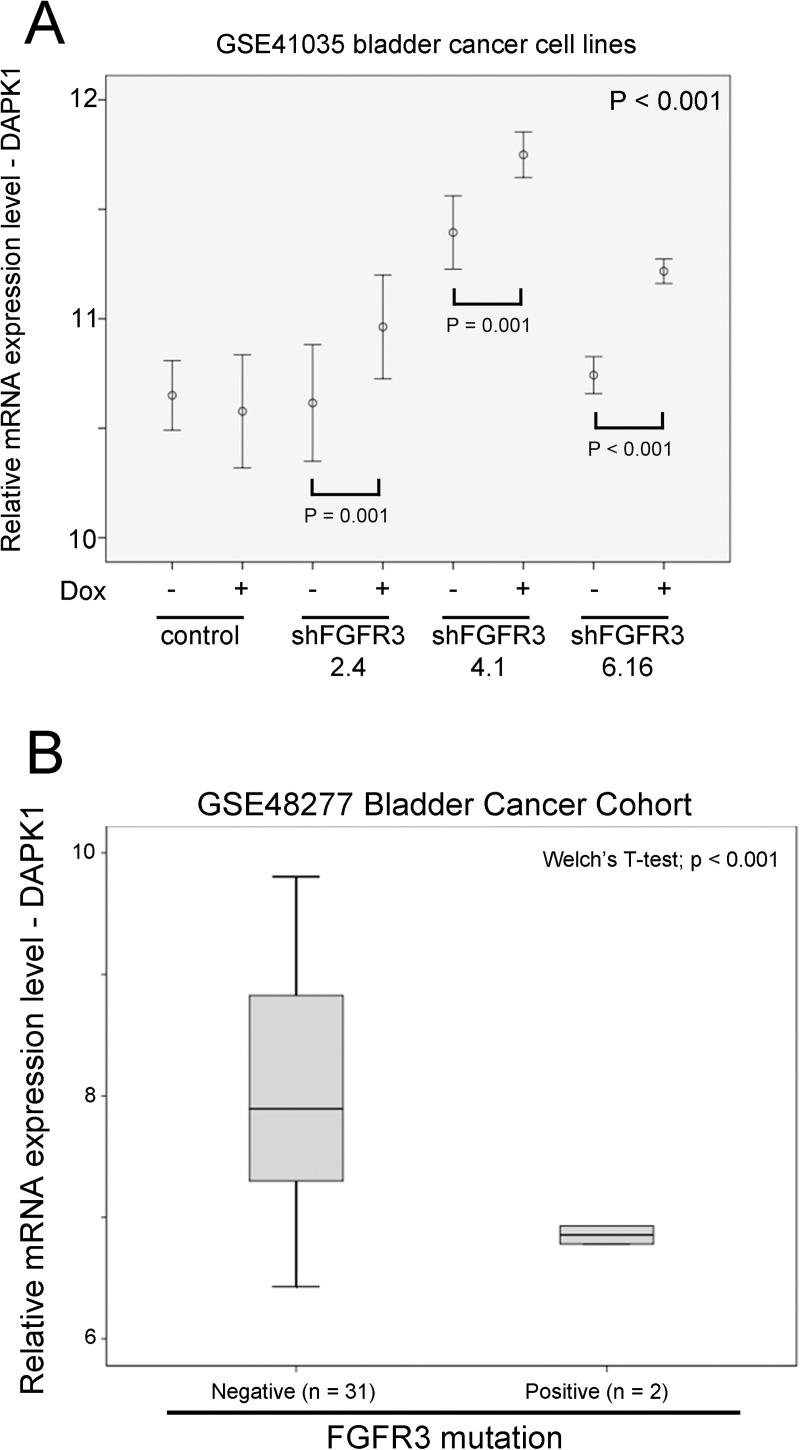
The association between FGFR3 and mRNA expression of DAPK1. (A) An error bar plot for DAPK1 mRNA expression in bladder cancer cell lines, RT112, with different expression of FGFR3 modulated through doxycyclin inducible shRNA knockdown in GSE41035. (B) A box plot for DAPK1 mRNA expression against FGFR3 mutational status in bladder cancer specimens with low level expression of FGFR3 mRNA.

Aberrant activation of FGFR3 by overexpression of activation mutation is common in bladder cancer and it has been shown to modulate lipid metabolism in order to maintain tumor growth and survival [20]. Using microarray data generated from bladder cancer cell line, RT112, with inducible knockdown of FGFR3 from three different shFGFR3 sequences, we found that DAPK1 expression was upregulated by knockdown of FGFR3 in inducible conditions for all the three different shFGFR3 sequences (p < 0.001; Fig 4A). In GSE48277, where both FGFR3 expression and mutational status were available, we further analyzed the relationship between FGFR3 mutation and expression, and DAPK1 expression. Although the expression level of DAPK1 was not significantly different between FGFR3 wild-type and mutation group when FGFR3 was expressing at a high level, DAPK1 expression was significantly lower in the specimens with FGFR3 activation mutation compared to those without the mutation when FGFR3 was expressing at a low level (p < 0.001; Fig 4B). Our results suggest that in a background of low FGFR3 expression, its activating mutation may play a significant role in suppressing the expression of DAPK1 in bladder cancer.

### The identification of DAPK1 co-regulated gene targets

**Fig 5:**
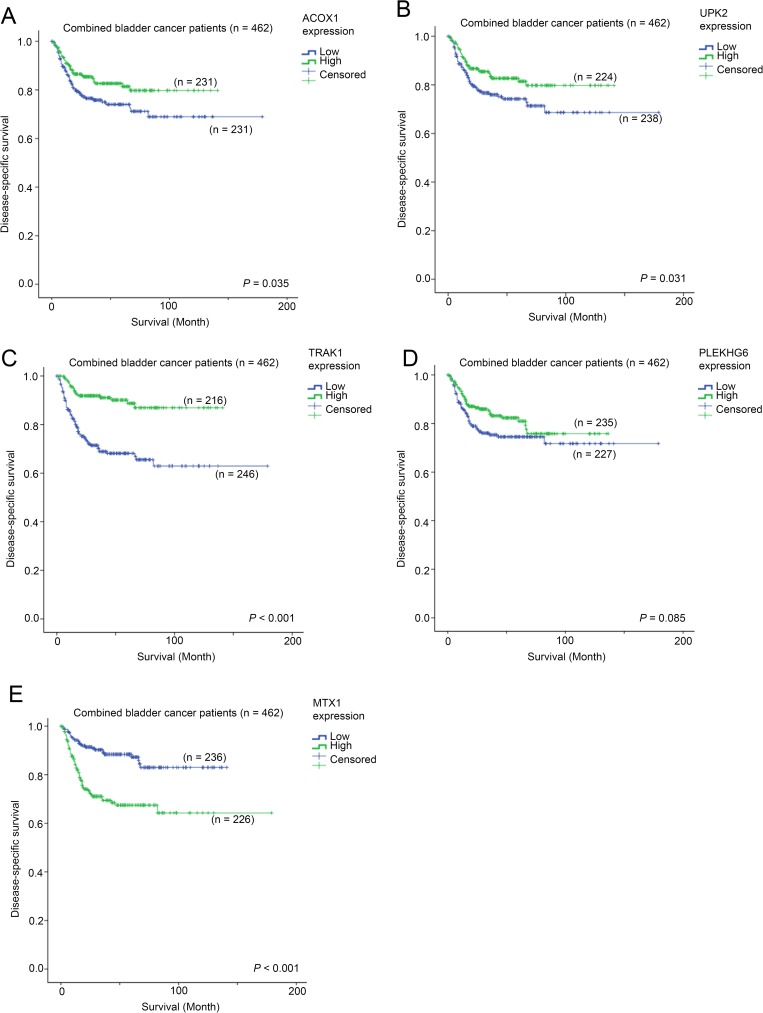
The association between mRNA expression of DAPK1-associated genes survival in combined bladder cancer cohort. Kaplan-Meier plots for survival for bladder cancer patients with different levels of mRNA expression of (A) ACOX1, (B) UPK2, (C) TRAK1, (D) PLEKHG6 and (E) MTX1.

By stratifying the specimens based on their DAPK1 expression levels, we compared the expression patterns of genes and identified genes that are differentially expressed between DAPK1-high and DAPK1-low subgroups. The top 5 genes that are consistently differentially expressed in the 3 bladder cancer datasets, GSE13507, GSE32894 and GSE48277, were further analyzed. As shown in Tables 1–4, the expression levels of ACOX1, UPK2, TRAK1 and PLEKHG6 were significantly positively correlated with DAPK1 expression level (p < 0.001), while the expression level of MT1X was significantly negatively correlated with DAPK1 expression level (p < 0.001). By univariate cox-regression analysis, we found that patients with a high level expression of ACOX1, UPK2 and TRAK1, had a 36% (HR = 0.64, 95% CI = 0.42–0.97, p = 0.036; Fig 5A), 37% (HR = 0.63, 95% CI = 0.42–0.96, p = 0.033; Fig 5B) and 82% (HR = 0.28, 95% CI = 0.17–0.46, p < 0.001; Fig 5C) reduction in risk of death or disease-related event, respectively, in the combined dataset. A high expression of PLEKHG6 was also associated with shorter survival time but it was not significant (Fig 5D). On the other hand, patients with a high level expression of MT1X had a significant 2.8-fold increase in risk of death or disease-related event (HR = 2.83, 95% CI = 1.81–4.41, p < 0.001; Fig 5E) in the combined dataset.

**Table 1:** GSE13507 dataset (n = 256) DAPK1 Correlation.

			DAPK1	ACOX1	UPK2	TRAK1	PLEKHG6	MT1X
Spearman's rho	DAPK1	Correlation Coefficient	1.000	.471^**^	.526^**^	.546^**^	.478^**^	-.519^**^
		*P* value	.	< 0.001	< 0.001	< 0.001	< 0.001	< 0.001
	ACOX1	Correlation Coefficient	.471^**^	1.000	.640^**^	.629^**^	.487^**^	-.275^**^
		*P* value	< 0.001	.	< 0.001	< 0.001	< 0.001	< 0.001
	UPK2	Correlation Coefficient	.526^**^	.640^**^	1.000	.660^**^	.521^**^	-.467^**^
		*P* value	< 0.001	< 0.001	.	< 0.001	< 0.001	< 0.001
	TRAK1	Correlation Coefficient	.546^**^	.629^**^	.660^**^	1.000	.459^**^	-.428^**^
		*P* value	< 0.001	< 0.001	< 0.001	.	< 0.001	< 0.001
	PLEKHG6	Correlation Coefficient	.478^**^	.487^**^	.521^**^	.459^**^	1.000	-.467^**^
		*P* value	< 0.001	< 0.001	< 0.001	< 0.001	.	< 0.001
	MT1X	Correlation Coefficient	-.519^**^	-.275^**^	-.467^**^	-.428^**^	-.467^**^	1.000
		*P* value.	< 0.001	< 0.001	< 0.001	< 0.001	< 0.001	.

**. Correlation is significant at the 0.01 level (2-tailed).

**Table 2:** GSE32548 dataset (n = 131) DAPK1 Correlation.

			DAPK1	ACOX1	UPK2	TRAK1	PLEKHG6	MT1X
Spearman's rho	DAPK1	Correlation Coefficient	1.000	.589^**^	.589^**^	.521^**^	.558^**^	-.529^**^
		*P* value	.	< 0.001	< 0.001	< 0.001	< 0.001	< 0.001
	ACOX1	Correlation Coefficient	.589^**^	1.000	.617^**^	.447^**^	.533^**^	-.504^**^
		*P* value	< 0.001	.	< 0.001	< 0.001	< 0.001	< 0.001
	UPK2	Correlation Coefficient	.589^**^	.617^**^	1.000	.590^**^	.600^**^	-.658^**^
		*P* value	< 0.001	< 0.001	.	< 0.001	< 0.001	< 0.001
	TRAK1	Correlation Coefficient	.521^**^	.447^**^	.590^**^	1.000	.415^**^	-.634^**^
		*P* value	< 0.001	< 0.001	< 0.001	.	< 0.001	< 0.001
	PLEKHG6	Correlation Coefficient	.558^**^	.533^**^	.600^**^	.415^**^	1.000	-.485^**^
		*P* value	< 0.001	< 0.001	< 0.001	< 0.001	.	< 0.001
	MT1X	Correlation Coefficient	-.529^**^	-.504^**^	-.658^**^	-.634^**^	-.485^**^	1.000
		*P* value.	< 0.001	< 0.001	< 0.001	< 0.001	< 0.001	.

**. Correlation is significant at the 0.01 level (2-tailed).

**Table 3:** GSE32894 dataset (n = 308) DAPK1 Correlation.

			DAPK1	ACOX1	UPK2	TRAK1	PLEKHG6	MT1X
Spearman's rho	DAPK1	Correlation Coefficient	1.000	.592^**^	.618^**^	.573^**^	.582^**^	-.529^**^
		*P* value	.	< 0.001	< 0.001	< 0.001	< 0.001	< 0.001
	ACOX1	Correlation Coefficient	.592^**^	1.000	.657^**^	.501^**^	.573^**^	-.502^**^
		*P* value	< 0.001	.	< 0.001	< 0.001	< 0.001	< 0.001
	UPK2	Correlation Coefficient	.618^**^	.657^**^	1.000	.626^**^	.667^**^	-.595^**^
		*P* value	< 0.001	< 0.001	.	< 0.001	< 0.001	< 0.001
	TRAK1	Correlation Coefficient	.573^**^	.501^**^	.626^**^	1.000	.563^**^	-.641^**^
		*P* value	< 0.001	< 0.001	< 0.001	.	< 0.001	< 0.001
	PLEKHG6	Correlation Coefficient	.582^**^	.573^**^	.667^**^	.563^**^	1.000	-.522^**^
		*P* value	< 0.001	< 0.001	< 0.001	< 0.001	.	< 0.001
	MT1X	Correlation Coefficient	-.548^**^	-.502^**^	-.595^**^	-.641^**^	-.522^**^	1.000
		*P* value.	< 0.001	< 0.001	< 0.001	< 0.001	< 0.001	.

**. Correlation is significant at the 0.01 level (2-tailed).

**Table 4:** GSE48277 dataset (n = 142) DAPK1 Correlation.

			DAPK1	ACOX1	UPK2	TRAK1	PLEKHG6	MT1X
Spearman's rho	DAPK1	Correlation Coefficient	1.000	.710^**^	.649^**^	.677^**^	.584^**^	-.674^**^
		*P* value	.	< 0.001	< 0.001	< 0.001	< 0.001	< 0.001
	ACOX1	Correlation Coefficient	.710^**^	1.000	.626^**^	.629^**^	.562^**^	-.617^**^
		*P* value	< 0.001	.	< 0.001	< 0.001	< 0.001	< 0.001
	UPK2	Correlation Coefficient	.649^**^	.626^**^	1.000	.730^**^	.740^**^	-.691^**^
		*P* value	< 0.001	< 0.001	.	< 0.001	< 0.001	< 0.001
	TRAK1	Correlation Coefficient	.677^**^	.629^**^	.730^**^	1.000	.590^**^	-.754^**^
		*P* value	< 0.001	< 0.001	< 0.001	.	< 0.001	< 0.001
	PLEKHG6	Correlation Coefficient	.584^**^	.562^**^	.740^**^	.590^**^	1.000	-.577^**^
		*P* value	< 0.001	< 0.001	< 0.001	< 0.001	.	< 0.001
	MT1X	Correlation Coefficient	-.674^**^	-.617^**^	-.691^**^	-.754^**^	-.577^**^	1.000
		*P* value.	< 0.001	< 0.001	< 0.001	< 0.001	< 0.001	.

**. Correlation is significant at the 0.01 level (2-tailed).

**Fig 6:**
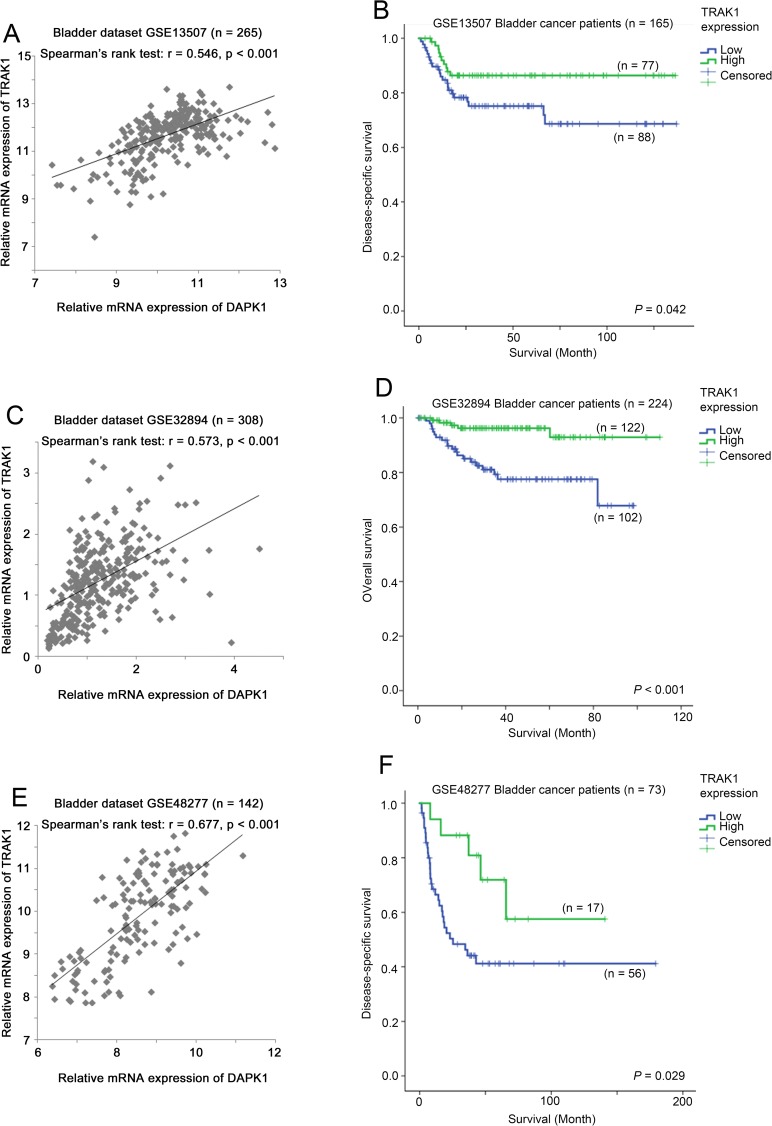
mRNA expression of TRAK1 in bladder cancer datasets. (A) A scatter plot showing the correlation between the expression levels of DAPK1 and TRAK1 in bladder cancer dataset GSE13507. (B) A Kaplan-Meier plot for patient survival with different level of TRAK1 expression in bladder cancer dataset GSE13507. (C) A scatter plot showing the correlation between the expression levels of DAPK1 and TRAK1 in bladder cancer dataset GSE32894. (D) A Kaplan-Meier plot for patient survival with different level of TRAK1 expression in bladder cancer dataset GSE32894. (E) A scatter plot showing the correlation between the expression levels of DAPK1 and TRAK1 in bladder cancer dataset GSE48277. (F) A Kaplan-Meier plot for patient survival with different level of TRAK1 expression in bladder cancer dataset GSE48277.

As shown in Fig 6, we found that the expression levels of DAPK1 and TRAK1 were positively significantly correlated with each other and that a low level expression of TRAK1 was associated with a poorer survival in the three bladder cancer patient cohorts. Our results show that TRAK1 expression was significantly associated with both DAPK1 expression and survival consistently in all the three bladder cancer patient cohorts and suggest that TRAK1 is a favourable prognostic marker in bladder cancer.

**Fig 7:**
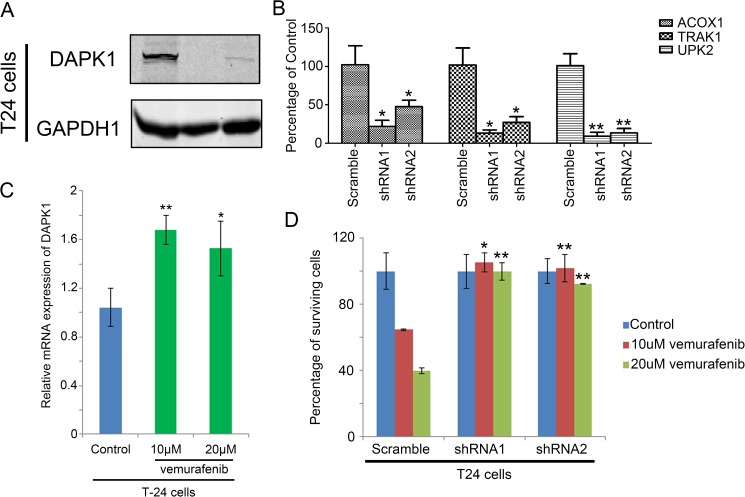
In vitro analysis for DAPK1 in bladder cancer. (A) Western blot analysis for DAPK1 in scramble and DAPK1 knockdown T24 cells. (B) Histogram showing the relative expression of ACOX1, TRAK1 and UPK2 in Scramble and DAPK1 knockdown T24 cells. (C) Histogram showing the relative mRNA expression of DAPK1 in control or vemurafenib-treated T24 cells. (D) Histogram showing the percentage of cells survived in control and vemurafenib treated scramble and DAPK1 knockdown T24 cells.

To confirm the findings from bladder cancer datasets, an in vitro experiment was carried out. DAPK1 was knocked down in a bladder cancer cell lines, T24 (Fig 7A), and mRNA expression levels of these genes were measured. As shown in Fig 7B, we found that in T24 cells, ACOX1, TRAK1 and UPK2 were significantly lower in DAPK1 knockdown cells compared to the Scramble control cells. Our results suggest that ACOX1, UPK2 and TRAK1 may be potential downstream targets of DAPK1.

### The identification of small molecules that target DAPK1 gene signature

Among the top 10 small molecules identified by connectivity mapping that can reverse the DAPK1-associated gene signature (Table 5; the full results of connectivity mapping is listed in S3 Table), vemurafenib and trametinib have been shown to confer anti-tumor activity in melanoma via inhibition of the Braf/MEK/ERK pathway [25–27]. We tested our hypothesis using T24 cell line and treated it with vemurafenib as Braf is a more upstream component of the Braf/MEK/ERK pathway, while this molecule has been shown to be an effective treatment for melanoma patients in a Phase III study [27]. Our results demonstrated that DAPK1 expression was upregulated by vemurafenib, in line with what we have shown by connectivity mapping (Fig 7C). Indeed, from the in vitro analysis, we also found that DAPK1 knockdown T24 cells were less sensitive to treatment of vemurafenib compared to scramble control cells (Fig 7D). Our results suggest that inhibition of Braf may be a potential therapeutic approach for patients with bladder cancer, while this approach may be more effective in treating bladder cancer with a high level expression of DAPK1.

**Table 5:** The top 10 small molecules identified via connectivity mapping that could reverse the DAPK1-associated gene expression.

Compound	p-value	Perturb stability
Pronestyl	< 0.001	1
Sulfisoxazole	< 0.001	1
Protriptyline hydrochloride	< 0.001	1
PLX4032 (vemurafenib)	< 0.001	1
Furosemide	< 0.001	1
Toremifene	< 0.001	1
Priscoline	< 0.001	0.933
Dantrolene	< 0.001	0.933
GSK1120212 (trametinib)	< 0.001	0.867
Cefepime hydrochloride	< 0.001	0.8

## Discussion

In this study, we have shown that DAPK1 was downregulated in bladder cancer with high T-stage in 4 independent bladder cancer patient cohorts (n = 743), each with more than 130 patient specimens, and a low level expression of DAPK1 was correlated with poor prognosis in 3 independent bladder cancer patient cohorts with patient survival status available (n = 462). Our results suggest that reduced expression of DAPK1 may lead to bladder cancer with a more aggressive phenotype. In addition, we found that FGFR3 knockdown downregulated DAPK1 in bladder cancer cell line, and TREK1 is coregulated with DAPK1 and a low level expression of TREK1 was also correlated with poor survival in 3 independent bladder cancer patient cohorts. Importantly, we identified via connectivity mapping that inhibition of Braf/MEK/ERK pathway may be a potential therapeutic approach for bladder cancer with reduced expression of DAPK1.

DAPK1 promoter methylation has been detected in majority of bladder cancer specimens [11, 13], transcriptional dyeregulation could be one of the major steps for DAPK1 downregulation in bladder cancer. In this study, we have found that DAPK1 mRNA expression was downregulated in bladder cancer with high T-stage and with shorter survival time, whether the downregulation of DAPK1 in these specimens was solely by promoter hypermethylation is unknown, nonetheless, the results suggest that reduced expression of DAPK1 play an important role in bladder cancer progression.

Fibroblast growth factor receptor 3 (FGFR3) has been shown to be a druggable target in bladder cancer [28], with its activating mutation being associated with recurrence [29]. In the present study, we have found that DAPK1 was downregulated upon inducible knockdown of FGFR3 suggesting that DAPK1 may act as a downstream factor of FGFR3 signaling. Although we found that in a low FGFR3 expression level background, DAPK1 expression was significantly lower in specimens with activating mutation of FGFR3, the results must interpret with caution since the number of analyzable specimens was relatively small. Further in vitro study is required to establish the functional role of DAPK1 in FGFR3-activating bladder cancer.

Little is known about the role of TRAK1 in bladder cancer. TRAK1 has been shown to play a role in endosome-to-lysosome trafficking and GABA(A) receptor homeostasis in hypertonia, which is observed in human neurological diseases [30, 31]. TRAK1 has been shown to be a promising diagnostic marker for gastric cancer [32] and a prognostic marker in colorectal cancer [33]. In the present study, we have found that TRAK1 was co-regulated with DAPK1 and was a favorable prognostic marker in bladder cancer. Whether TRAK1 plays a different role in bladder cancer progression compared to gastric and colorectal cancer should be further studied.

Mutation of Braf gene in bladder cancer is an infrequent event [34], however, Ras mutation, which act upstream of Braf, or Ras pathway has been shown to play an important role in bladder cancer tumorigenesis [35], while MEK inhibitor, PD-0325901, has been shown to suppress bladder tumor growth in a patient-derived xenograft model [36] and the inhibition of Raf/MEK/ERK pathway through Cholera toxin also resulted in growth arrest in cell line model [37]. In this study, we found that vemurafenib, a Braf inhibitor, and trametinib, a MEK inhibitor, both could reverse DAPK1-associated gene expression signature, by connectivity mapping. The results have been further confirmed in a bladder cancer cell line, in which treatment of vemurafenib resulted in upregulation of vemurafenib. Interestingly, knockdown of DAPK1 in T24 cells led to reduced sensitivity towards vemurafenib. Our results prime others to test whether DAPK1 expression could be a potential predictive marker for Raf/MEK/ERK pathway inhibition.

In conclusion, our data show that DAPK1 could be a suppressor in bladder cancer progression, and suggest that DAPK1 could be a potential biomarker and therapeutic target in bladder cancer.

## Supporting information

S1 Fig Flow Chart. Extraction of clinical and microarray gene expression data from bladder cancer patient datasets.
(TIF)

S1 Table shRNA sequences of scramble and targeting DAPK. (DOCX)

S2 Table Summary of Primer Sequences and Product Size Used in the PCR procedure. (DOCX)

S3 Table Connectivity Mapping. (XLSX)
