# Supplementary figures and images for: The prognostic significance of DAPK1 in bladder cancer
Source: PLoS One. 2017 Apr 7;12(4):e0175290. doi: 10.1371/journal.pone.0175290 (PMC5384764; doi:10.1371/journal.pone.0175290)

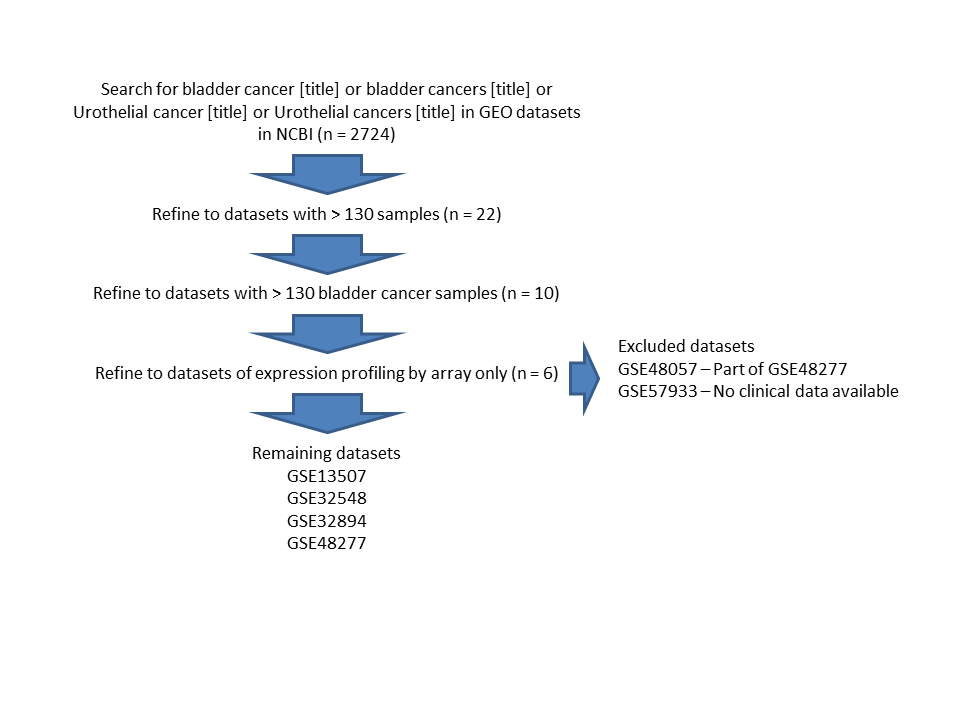

Supplement: S1 Fig — Extraction of clinical and microarray gene expression data from bladder cancer patient datasets. (TIF) [file pone.0175290.s001.tif]
