# Supplementary material for: The prognostic significance of DAPK1 in bladder cancer
Source: PLoS One. 2017 Apr 7;12(4):e0175290. doi: 10.1371/journal.pone.0175290 (PMC5384764; doi:10.1371/journal.pone.0175290)
Supplement: S1 Table — (DOCX) [file pone.0175290.s002.docx]

**S1 Table. shRNA sequences of scramble and targeting DAPK**

| scramble | CCGGGCGCGATAGCGCTAATAATTTCTCGAGAAATTATTAGCGCTATCGCGCTTTTT |
| --- | --- |
| shRNA1 | TRCN0000000983，CCGGCCACGTCGATACCTTGAAATTCTCGAGAATTTCAAGGTATCGACGTGGTTTTT |
| shRNA2 | TRCN0000273223，CCGGTACCTTGCTTCTTACTGATAACTCGAGTTATCAGTAAGAAGCAAGGTATTTTTG |
