# Supplementary material for: The prognostic significance of DAPK1 in bladder cancer
Source: PLoS One. 2017 Apr 7;12(4):e0175290. doi: 10.1371/journal.pone.0175290 (PMC5384764; doi:10.1371/journal.pone.0175290)
Supplement: S2 Table — (DOCX) [file pone.0175290.s003.docx]

**S2 Table. Summary of Primer Sequences and Product Size Used in the PCR procedure**

| Gene name | Sequences（5´-3´） | Product Size |
| --- | --- | --- |
| DAPK1 | F: AATCCTAGACGTGGTCCGGTAT | 155 bp |
|  | R:GTCCTCGGTGCGTATCCTTTCG |  |
| ACOX1 | F: GAGCCTCTGGATCTTCACTT | 172 bp |
|  | R: TGGGTTTCAGGGTCATACGT |  |
| TRAK1 | F: GGAGGAGAGTGAGCCCGAGT | 156 bp |
|  | R: AGCTGGCTGGCCTCGGATC |  |
| UPK2 | F: GCAATGCCACACTGATGGTC | 107bp |
|  | R: GTCCACCACACTCACCAGTT |  |
| GAPDH RT PCR | F: CGGAGTCAACGGATTTGGTCGTAT | 304 bp |
|  | R: AGCCTTCTCCATGGTGGTGAAGAC |  |

F, forward primer; R, reverse primer.
